# Contralateral Cruciate Survival in Dogs with Unilateral Non-Contact Cranial Cruciate Ligament Rupture

**DOI:** 10.1371/journal.pone.0025331

**Published:** 2011-10-05

**Authors:** Peter Muir, Zeev Schwartz, Sarah Malek, Abigail Kreines, Sady Y. Cabrera, Nicole J. Buote, Jason A. Bleedorn, Susan L. Schaefer, Gerianne Holzman, Zhengling Hao

**Affiliations:** 1 Comparative Orthopaedic Research Laboratory, and the Department of Surgical Sciences, School of Veterinary Medicine, University of Wisconsin-Madison, Madison, Wisconsin, United States of America; 2 Department of Surgery, VCA West Los Angeles Animal Hospital, Los Angeles, California, United States of America; 3 California Animal Hospital, Los Angeles, California, United States of America; Universidad Europea de Madrid, Spain

## Abstract

**Background:**

Non-contact cranial cruciate ligament rupture (CrCLR) is an important cause of lameness in client-owned dogs and typically occurs without obvious injury. There is a high incidence of bilateral rupture at presentation or subsequent contralateral rupture in affected dogs. Although stifle synovitis increases risk of contralateral CrCLR, relatively little is known about risk factors for subsequent contralateral rupture, or whether therapeutic intervention may modify this risk.

**Methodology/Principal Findings:**

We conducted a longitudinal study examining survival of the contralateral CrCL in client-owned dogs with unilateral CrCLR in a large baseline control population (n = 380), and a group of dogs that received disease-modifying therapy with arthroscopic lavage, intra-articular hyaluronic acid and oral doxycycline (n = 16), and were followed for one year. Follow-up in treated dogs included analysis of mobility, radiographic evaluation of stifle effusion and arthritis, and quantification of biomarkers of synovial inflammation. We found that median survival of the contralateral CrCL was 947 days. Increasing tibial plateau angle decreased contralateral ligament survival, whereas increasing age at diagnosis increased survival. Contralateral ligament survival was reduced in neutered dogs. Our disease-modifying therapy did not significantly influence contralateral ligament survival. Correlative analysis of clinical and biomarker variables with development of subsequent contralateral rupture revealed few significant results. However, increased expression of T lymphocyte-associated genes in the index unstable stifle at diagnosis was significantly related to development of subsequent non-contact contralateral CrCLR.

**Conclusion:**

Subsequent contralateral CrCLR is common in client-owned dogs, with a median ligament survival time of 947 days. In this naturally occurring model of non-contact cruciate ligament rupture, cranial tibial translation is preceded by development of synovial inflammation. However, treatment with arthroscopic lavage, intra-articular hyaluronic acid and oral doxycycline does not significantly influence contralateral CrCL survival.

## Introduction

Non-contact cranial cruciate ligament rupture (CrCLR) is an important cause of lameness in the dog that incurs substantial annual health-care costs [1]. It is now widely recognized that mid-substance rupture of the CrCL often occurs during normal activity, with a high incidence of bilateral rupture at the time of initial clinical presentation in the range of 11–17% [2]–[4]. The CrCL in dogs is anatomically equivalent to the anterior cruciate ligament (ACL) in human beings, and the dog is a widely used model for research into ACL biology and repair.

A number of studies have examined the incidence of subsequent contralateral CrCLR in dogs diagnosed with unilateral non-contact CrCLR at initial presentation. Analysis of this risk has usually been reported as an incidence after surgery (percentage of patients within the cohort). This risk is in the range of 22–54% at 6 to 17 months of diagnosis [2], [3], [5]–[7]. One of the limitations of this approach to data analysis is that it yields little information on the pattern of subsequent contralateral rupture. It has been our clinical impression that individual dogs appear at particularly high risk of subsequent contralateral CrCLR, whereas other dogs are protected from the trait. Estimation of ligament survival over time would provide more detailed information on the pattern of subsequent contralateral CrCLR.

In the past, development of stifle arthritis was thought to occur secondary to development of joint instability associated with progressive CrCLR. However, this perspective may not be correct, since development of stifle synovitis is an early event that can often be found before fraying of the cruciate ligaments becomes arthroscopically detectable in dogs with incipient disease [8]. Ligament damage typically involves both the CrCL and the caudal cruciate ligament [9]. Development of stifle synovitis also increases the risk of subsequent contralateral CrCLR in dogs [10].

The presence of synovitis in stifle joints with incipient CrCLR suggests that immune-mediated joint degeneration is a factor in the non-contact CrCLR mechanism. The cruciate ligaments are wrapped in synovium [11]; synovial vasculature has a blood-CrCL barrier, analogous to the blood-brain barrier [12], suggesting that inflammatory changes within synovium and synovial fluid have profound effects on cruciate ligament tissue metabolism. It is also known that chronic synovitis induces marked deterioration in CrCL structural properties in a rabbit model [13]. Histologic features of lymphoplasmacytic synovitis are present in stifle joints of 51–67% affected dogs at the time of initial diagnosis [10], [14]. In affected dogs, inflammatory cell populations within stifle synovium are usually mononuclear, and include T lymphocytes, B lymphocytes, major histocompatibility complex (MHC) class II^+^ dendritic cells, and activated macrophages, expressing tartrate-resistant acid phosphatase (TRAP) [15]–[18]. TRAP^+^ mononuclear cells are not found in normal stifle synovium of dogs [17].

At the present time, there are two hypotheses regarding the mechanism that leads to development of inflammatory stifle arthritis and eventual CrCLR: (1) cruciate fiber rupture is a consequence of a primary synovitis [8], and (2) intrinsic and extrinsic factors induce fiber rupture, with subsequent induction of synovitis by ligament matrix neoepitopes [19]–[21]. The immunologic trigger for synovitis for the first hypothesis is not known, although the presence of bacterial material within the stifle has been associated with the condition [22]–[24].

If development of synovitis has a significant role in the pathogenesis of progressive non-contact rupture of the cruciate ligament complex, particularly the CrCL, then disease-modifying medical therapy may block progressive stifle joint degeneration in dogs with incipient disease, and lead to a reduction in the incidence of non-contact cruciate rupture over time. In this regard, evaluation of the status of the contralateral CrCL over time in dogs with unilateral CrCLR would be one model that could be used to address this question.

The first objective of the study was to conduct a survival analysis of time to contralateral CrCLR in a large population of dogs with unilateral CrCLR presented for surgical treatment, to determine the pattern of subsequent contralateral CrCLR that is typically found in affected dogs. We hypothesized that within a large population of affected dogs, some dogs would appear protected from the risk of subsequent contralateral rupture. A secondary objective of this study was to determine whether treatment of a group of dogs with a provisional disease-modifying therapy for arthritis might ameliorate risk of subsequent contralateral rupture, and whether a biomarker for subsequent contralateral rupture risk could be identified.

## Materials and Methods

### Dogs

In order to determine the pattern of subsequent contralateral CrCLR in affected dogs, we performed a survival analysis of three populations of dogs from different regions of the USA; data from two of these cohorts has been previously published with limited analysis on contralateral CrCL survival [2], [3]. Dogs with unilateral CrCLR that were treated surgically were studied.

In *Group 1*, a cohort of 125 dogs [2] presented to the Department of Surgery, VCA West Los Angeles Hospital, Los Angeles, CA between 2000 and 2006 was studied. In *Group 2*, a cohort of 84 Labrador retrievers [3] presented to the Animal Medical Center, New York, NY and Dallas Veterinary Surgical Center, Dallas, TX between 2000 and 2003 was studied. In *Group 3*, a cohort of 171 dogs was studied at the University of Wisconsin-Madison, School of Veterinary Medicine, Veterinary Medical Teaching Hospital, Madison, WI. Initially, the medical records of all dogs (298 dogs) that received surgical treatment during the period 2007–2009 were reviewed for inclusion in the survival analysis. Of these records, 51 were excluded because of incomplete data or were lost to follow-up, 16 were excluded because a disease-modifying treatment with intra-articular hyaluronic acid and doxycycline was provided (see below), and 60 were excluded because the dog was found to have bilateral CrCLR on initial examination. The remaining 171 records were included in the study and examined in detail. In *Group 4*, a cohort of 16 dogs with unilateral CrCLR rupture and clinical and radiographic evidence of incipient non-contact cruciate rupture in the contralateral stifle were studied. Dogs presented to the University of Wisconsin-Madison, School of Veterinary Medicine, Veterinary Medical Teaching Hospital, Madison, WI [8] were studied, after treatment with a single intra-articular injection of hyaluronic acid (HA) and a course of oral doxycycline. Doxycycline has been previously reported to have disease-modifying effects on arthritic joints in human beings and dogs [25]–[27]. All procedures at the University of Wisconsin-Madison were conducted with the approval of the Animal Care & Use Committee, School of Veterinary Medicine, University of Wisconsin-Madison (V1070).

### Medical records review

For inclusion in the analysis, CrCLR was confirmed during surgical treatment, with clinical follow-up of a least one year for censored cases. Breed, age, bodyweight, and gender were obtained from the medical record. Pre-operative tibial plateau angle (TPA) in the index stifle was also recorded [2], [3] in all groups. If the dog developed a subsequent contralateral CrCLR during the study period, the time interval to contralateral CrCLR was recorded in days as a complete case. For dogs that did not experience a contralateral CrCLR, the time interval to clinical or telephone follow-up was recorded, as a censored case.

Follow-up information was obtained from the medical record, by telephone conversation with the owner, or telephone conversation with the attending clinician, if surgery for subsequent contralateral CrCLR had been performed at a different hospital, as previously described [2], [3]. In the Groups 2 and 3, a standard questionnaire was used to obtain telephone follow-up, as previously described [3].

### Provisional disease-modifying therapy for synovitis/arthritis

Initial clinical, radiographic, and arthroscopic findings in the cohort of dogs in Group 4 have been previously reported [8]. Inclusion criteria for this cohort of dogs included 1) pelvic limb lameness; 2) clinical and radiographic signs of synovial effusion and arthritis in both stifles; 3) stifle instability in the index stifle; 4) no clinically detectable instability in the contralateral stifle based on palpation and stress radiographic examination [8]; 5) no history of traumatic injury; and 6) no previous stifle surgery.

Age, weight, gender, and history were recorded. Bilateral orthogonal stifle radiographs were made immediately before surgery, at 10 weeks after surgery, and at long-term follow-up at least 12 months after surgery. Radiographs were graded for the following parameters: “overall disease severity” (0–3), “joint effusion” (0–2), “osteophytosis” (0–3) and “intraarticular mineralization” (0–2) [28]. Radiographic grading was performed by a single observer (PM). In addition during the initial visit and at long-term follow-up, stress radiographs of the contralateral stifle were made [8]. Dogs were positioned in lateral recumbency, with the affected stifle resting on the x-ray table. The x-ray beam was centered over the stifle joint and collimated to include both the tibial and femoral diaphysis. A standard lateral view was taken with the joint in approximately 90–110 degrees of flexion (neutral view). For the tibial compression view, the stifle was maintained as the same angle of flexion, and manual stress was applied to the metatarsals to flex the hock joint maximally (stress view). Cranial tibial translation was then measured [8] by a single observer (ZS).

During surgical stabilization of the index stifle with tibial plateau leveling osteotomy (TPLO), arthoscopic examination of the unstable index and contralateral stable stifle joints was performed [8]. Pain management was supervised by a board-certified veterinary anesthesiologist. Dogs were premedicated, anesthetized, and administered an epidural or regional nerve block before surgery. No intra-articular anesthetic agents were used. Immediately after surgery, 20 mg of HA (Hylartin V, Pfizer Animal Health, New York, NY) was injected into each stifle and doxycycline was given orally at 5 mg/kg b.i.d. for 10 weeks.

### Clinical follow-up of HA/doxycycline-treated dogs

Follow-up visits were performed at 10 weeks and at least one year, except for one dog that died shortly before one year after surgery. At the long-term recheck each owner was asked to complete a specific outcome measure questionnaire based on the “Liverpool Osteoarthritis in Dogs” (LOAD) questionnaire [29]. In completing the LOAD questionnaire, owners were asked to score the function of the dog in the period immediately before surgery and then again in the period immediately before the long-term follow-up visit. The total possible score ranged from 0–52, with 0 being normal.

Force-plate analysis-of-gait was also performed at long-term follow-up. Peak vertical force (F_z_) and vertical impulse (VI) normalized to body weight were obtained [30]. A biomechanical platform that measures 3-dimensional forces and impulses (OR6-6-1000 Biomechanics Platform with SGA6-4 Signal Conditioner/Amplifier, Advanced Mechanical Technologies Inc., Newton, MA) was used. The force plate was connected to a commercially available satellite data acquisition system (Sharon Software Inc., Dewitt, MI). The velocity of each trial was measured. At least 5 successful trials were obtained from each dog, other than one dog in which only 4 trials were obtained. A successful trial was defined by a thoracic limb hitting the plate followed by the ipsilateral pelvic limb within the predetermined velocity range of 1.1 to 1.8 m/s and an acceleration range of −0.5 m/s^2^ to +0.5 m/s^2^.

### Collection of peripheral blood and joint tissue specimens from HA/doxycycline-treated dogs

A peripheral blood sample was collected into a vacutainer tube (BD Vacutainer™ CPT™, Becton Dickinson, Franklin Lakes, NJ) during each visit. Peripheral blood mononuclear cells (PBMC) were subsequently isolated by centrifugation. During surgery, synovial membrane and synovial fluid specimens were collected bilaterally. Synovium was collected from the lateral suprapatellar region of the stifle at the site of the egress portal using aseptic technique [8]. Synovial fluid was also obtained bilaterally at both the 10 week and at long-term follow-up visits using aseptic technique and needle aspiration under sedation. Joint tissue specimens were then transported to a laboratory that was not used for routine bacteriological research. Specimens of synovium were divided in a laminar flow hood and portions of each biopsy were used for estimation of bacterial load and for histology. Synovial fluid cells were isolated by centrifugation. Synovial membrane, synovial fluid cells, and PBMC were stored at −80C until analyzed.

### Determination of bacterial load by 16S rRNA real-time PCR in HA/doxycycline-treated dogs

DNA was isolated from synovium and synovial fluid and bacterial load was estimated in each sample using two broad-ranging standard primer sets optimized for detection of complementary genera of bacteria [24], [31]–[33]. PCR reactions were carried out in a final volume of 25 µl using the TaqMan PCR core reagent kit (Applied Biosystems, Foster City, CA). The master mixture was filtered using a Centriprep YM-100 filter (Millipore, Billerica, MA), and then 1 µl of total DNA was added. All PCR reactions were performed in triplicate. To estimate bacterial copy number, the threshold cycle (C_t_) of each sample was analyzed against a standard curve with serial 10-fold dilution of DNA from 10 ng to 10 fg, using DNA derived from a rapidly growing aerobic organism previously identified in canine joints (*Stenotrophomonas maltophilia* ATCC #13637, Manassas, VA) [24]. To prevent contamination, all work was performed within a laminar flow cabinet treated with UV light. PCR amplification was performed in a different room from DNA extraction. Aliquots from known negative samples were used as an extraction control in each PCR run, in addition to negative and positive controls. PCR runs were limited to no more than eight samples in order to minimize the risk of handling errors during the assay.

### Quantitative reverse-transcriptase-polymerase chain reaction (qRT-PCR) of joint tissues from HA/doxycycline-treated dogs

cDNA was generated from 0.2–1 µg of total RNA isolated from synovium and synovial fluid as previously described [24]. qRT-PCR was performed using standard methods and SYBR green methodology using a Bio-Rad thermocycler (MyiQ and IQ-SYBR Green Supermix, Bio-Rad, Hercules, CA). Oligonucleotide primers for the following genes: variable region of the beta chain of the T lymphocyte antigen receptor (TCR Vβ), CD3ε, TRAP, interleukin-17 (IL-17), IL-10, IL-4, interferon-γ (IFN-γ), and tumor necrosis factor-α (TNF-α) (Table 1). The TCR complex consists of either a TCR α/β or γ/δ heterodimer co-expressed at the cell surface with invariant subunits (γ, δ, ε, η, ζ) of CD3 [34], [35]. Primers were designed from known canine gene sequences or regions of homology between the specific genes of other higher mammals. The 18S rRNA gene was used as the housekeeping gene. All PCR reactions were carried out as described [24]. Assays were validated by the use of a no template control. In addition, we tested the CD3ε and TCR Vβ primers using an immortalized Madin Darby canine kidney cell line and found no expression, as would be expected with genes that are only expressed in T lymphocytes.

**Table 1:** Canine oligonucleotide primers for quantitative real-time reverse-transcriptase polymerase chain reaction.

mRNA Targets	Primer Type	Olignonucleotides (5′ to 3′)	Amplicon Size (bp)	Sequence Reference
TCR-Vβ	Forward	ACAGTGACCCTGAGATGTTCCCTT	139	Laboratory of Dr. Muir
	Reverse	ATCTTGCCGGGATGTCTCCTTTGT		Dreitz et al. 1999 [65]
CD3å	Forward	CCAATTCCTTCCTACCCTTCAGAGGTAT	112	Laboratory of Dr. Muir
	Reverse	GAGCTGGAAAAACGGAAAGGT		Nash et al. 1991 [66]
TRAP	Forward	TGCTGGCCACGTACAAGGT	70	Laboratory of Dr. Muir
	Reverse	TCATCCTGAAGGTACTGCAGGTT		Partial canine sequence
IL-17	Forward	CACTCCTTCCGGCTAGAGAA	71	Maccoux et al. 2007 [54]
	Reverse	CACATGGCGAACAATAGGG		
IL-10	Forward	CGCTGTCACCGATTTCTTCC	78	Wang et al. 2007 [67]
	Reverse	CTGGAGCTTACTAAATGCGCTCT		
IL-4	Forward	CATCCTCACAGCGAGAAACG	83	Wang et al. 2007 [67]
	Reverse	CCTTATCGCTTGTGTTCTTTGGA		
IFN-γ	Forward	GCGCAAGGCGATAAATGAAC	82	Wang et al. 2007 [67]
	Reverse	CTGACTCCTTTTCCGCTTCCT		
TNF-α	Forward	GAGCCGACGTGCCAATG	79	Wang et al. 2007 [67]
	Reverse	CAACCCATCTGACGGCACTA		
18S rRNA	Forward	CGCCGCTAGAGGTGAAATTCT	100	Laboratory of Dr. Svaren
	Reverse	CGAACCTCCGACTTTCGTTCT		U. Wisconsin-Madison

**Note**: TCR-Vβ – variable region of the beta chain of the T lymphocyte antigen receptor; TRAP – tartrate-resistant acid phosphatase; IL – interleukin; IFN – interferon; TNF – tumor necrosis factor.

### Synovial membrane histology in HA/doxycycline-treated dogs

After collection, a portion of each synovial membrane specimen was fixed in Zamboni's fixative for 1–2 days at 4°C [36]. Frozen sections 10 µm thick were prepared [24] and stained with hematoxylin and eosin. Sections were also stained histochemically for TRAP using naphthol AS-BI phosphate as a marker for activated macrophages [17]. For each batch of slides, a negative control was prepared by omission of the naphthol AS-BI phosphate. Synovial tissue sections were scored subjectively for severity of synovial inflammation (J.A.B.) and numbers of TRAP^+^ synovial macrophages (P.M.) using a visual analog scale method [24].

### Statistical Analysis

Gehan's generalized Wilcoxon test and the Kaplan-Meier Product-Limit estimate were used for multivariate survival analysis to determine whether there were significant differences in contralateral ligament survival between groups of dogs (Groups 1–3), as a baseline control. Similarly, effects of gender or breed on contralateral ligament survival were also examined. The ten most common breeds in the study were analyzed (in order of prevalence in the cohort, Labrador retriever, Golden retriever, Rottweiler, German shepherd, Cocker spaniel, Boxer, Pitbull terrier, Beagle, Newfoundland, Mastiff). Cox's proportional hazard model was also used to determine whether age, gender, bodyweight, or pre-operative TPA had significant effects on contralateral CrCL survival. Time-dependent analysis was performed for dog age. Gehan's generalized Wilcoxon test and the Chi-square test were also used to determine whether contralateral CrCL survival was influenced by treatment with our provisional disease-modifying therapy.

For analysis of long-term clinical follow-up data, the Kolmolgorov-Smironv test was used to determine whether data approximated a normal distribution. If a dog developed contralateral CrCLR during the study period, then this stifle was excluded from the short- or long-term follow-up data sets. Bacterial copy number was normalized to the DNA concentration and the result was expressed as #/µg of DNA. For analysis of gene expression data, the threshold cycle (C_t_ values) obtained from the exponential region of the PCR amplification plot from duplicate trials were averaged together. Relative expression for each of the genes-of-interest was then calculated using standard curves [37]. PBMC gene expression was used as an internal control and the 18S rRNA gene was used as the housekeeping gene. Dependent data from index and contralateral limbs were analyzed using the Student's *t* test for paired data, or the Friedman ANOVA and Wilcoxon matched-pairs tests, as appropriate. Within each group and time point, the single sample Student's *t* test with a hypothesized mean of zero was used to determine whether expression of TCR Vβ, CD3ε, TRAP, IL-17, IL-4, IL-10, IFN-γ and TNF-α in synovial tissues and PBMC was different, after log-transformation of data. Results were considered significant at *p*<0.05.

Correlations between the development of subsequent contralateral CrCLR and a priori clinical, radiographic, arthroscopic, and synovial tissue laboratory analyses were examined using the Pearson and Spearman Rank correlation tests. Where relevant, a Bonferroni correction for comparisons across three time points, such that values of *p*<0.025 were considered significant.

## Results

### Contralateral Ligament Survival

**Figure 1:**
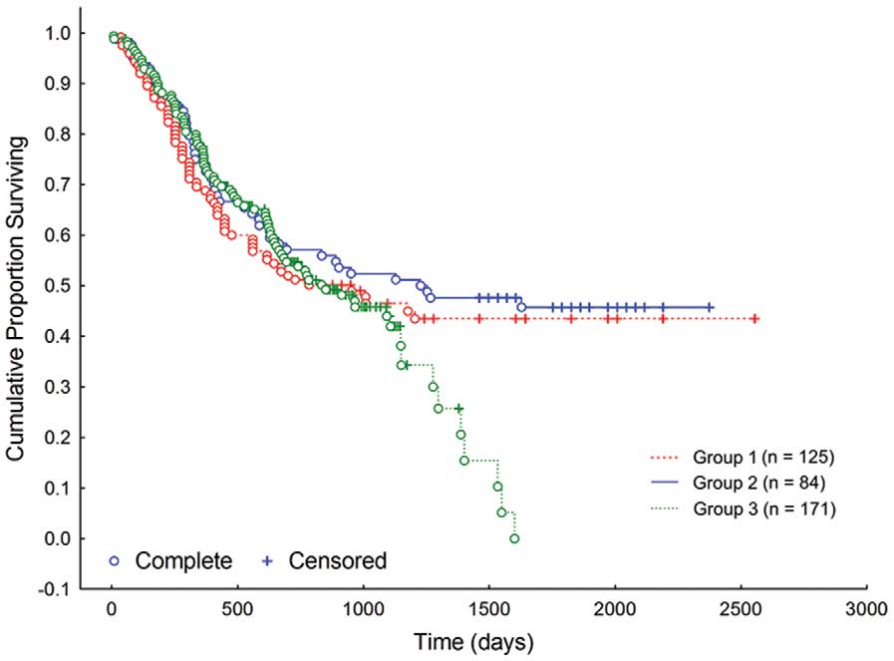
Contralateral cranial cruciate ligament (CrCL) survival. Kaplan-Meier plot for contralateral CrCL survival in three large populations of client-owned dogs. Overall median survival was 2.59 years. There was no significant difference in ligament survival between groups (*p* = 0.57). Complete – dogs that experienced contralateral CrCLR during the study period; Censored – dogs that did not experience contralateral CrCLR during the study period.

In Group 1, median survival time for the contralateral ligament was 809 days. Subsequent contralateral rupture occurred in 67 of 125 dogs (54%). In Group 2, median survival time was 1227 days. Subsequent contralateral rupture occurred in 45 of 84 dogs (54%). In Group 3, median survival time was 836 days. Subsequent contralateral rupture occurred in 92 of 171 dogs (54%) (**Fig. 1**). There were no significant differences between groups in the clinical survival of the contralateral CrCL (*p* = 0.57). Overall, median survival time for the contralateral ligament was 947 days or 2.59 years; 204 of 380 dogs (54%) developed subsequent contralateral rupture within the study period.

**Figure 2:**
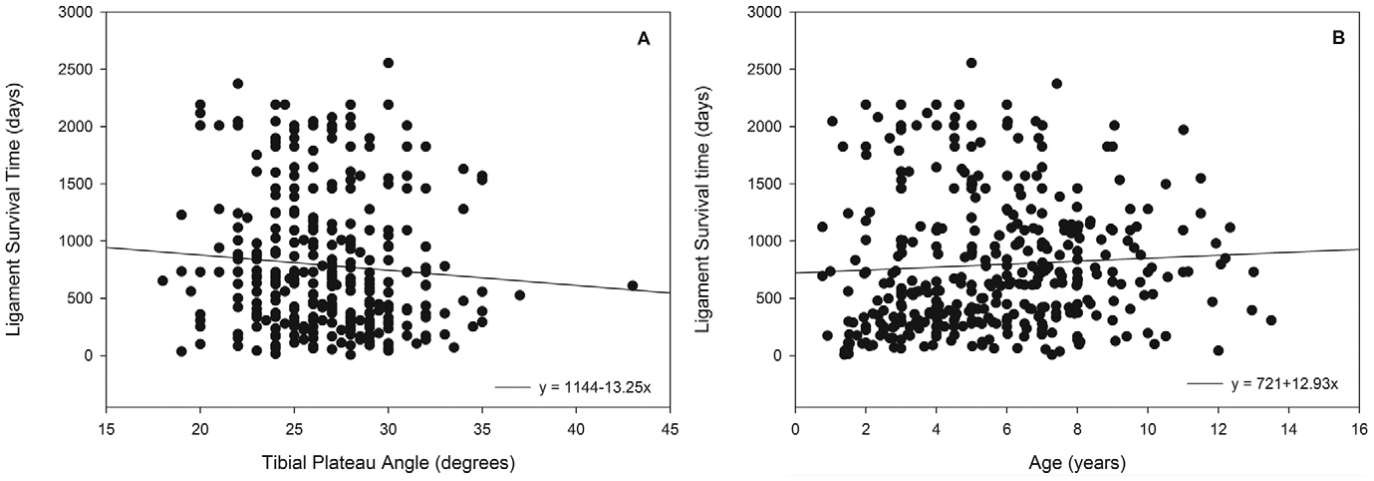
Tibial plateau angle (TPA) and age influence contralateral cranial cruciate ligament (CrCL) survival over time in dogs diagnosed with unilateral CrCL rupture. (**A**) Increasing TPA was associated with decreased contralateral CrCL survival, whereas increasing age at diagnosis was associated with increased contralateral CrCL survival (**B**). However, these effects appear minor, as the slope of the regression lines is small and data points are widely scattered.

In the combined cohort of 380 baseline dogs, dog age (*p*<0.05), TPA (*p*<0.05), and gender (*p*<0.05) all significantly influenced contralateral CrCL survival. The effect of body weight (*p* = 0.58) and breed (*p* = 0.62) were not significant. Increasing age was associated with increased contralateral CrCL survival, whereas increasing TPA was associated with decreased contralateral CrCL survival (**Fig. 2**). Median survival time of the CrCL was longer in male (950 days) and female (923 days) dogs, and shorter in both castrated males (708 days) and ovariohysterectomized females (845 days).

### Clinical follow-up of HA/doxycycline-treated dogs

Of the cohort of 16 dogs, all dogs were re-examined at 10 weeks after surgery. Thirteen dogs were re-examined at long-term follow-up at least one year after surgery. Telephone follow-up with the owner was obtained for the remaining 3 dogs. The 16 client-owned dogs with stifle arthritis and CrCLR consisted of the following breeds: Golden retriever (n = 4), Labrador retriever (n = 3), and individual dogs of other breeds: American bulldog, Blood hound, Chesapeake Bay retriever, German shorthair pointer, Great Dane, Mastiff, Samoyed, Siberian Husky, Saint Bernard. In this group of dogs, age was 5.2±2.5 years and body weight was 40.7±7.2 kg. Eight dogs were ovariohysterectomized females, 7 dogs were castrated males, and 1 dog was an intact male. Median duration of lameness was 19.5 weeks (range 1–156 weeks). None of the dogs had concurrent systemic disease, and all contralateral stifles were determined to be clinically stable by palpation under general anesthesia at diagnosis. At surgery, tearing of the medial meniscus was identified in the index stifle of 7 of 16 dogs (44%). No meniscal tears were found in the contralateral stable stifle.

All dogs were re-examined to evaluate clinical function and tibial osteotomy healing (10.2±1.3 weeks after surgery). By this time 1 of 16 dogs (6%) was diagnosed with contralateral CrCLR at 62 days after surgery. In the remaining 15 dogs, the contralateral stifle remained clinically stable. At long-term follow-up, an additional 4 dogs developed subsequent contralateral CrCLR, yielding a one-year incidence of 5 of 16 dogs (31%), which was not significantly different from the Group 3 dogs (*p* = 0.12). In two dogs with subsequent contralateral CrCLR, fraying of the CrCL was previously identified arthroscopically and estimated to involve 10% of the ligament, another dog was estimated to have a tear involving 30% of the ligament; in the two remaining dogs with subsequent contralateral CrCLR only minimal superficial fraying was observed.

**Figure 3:**
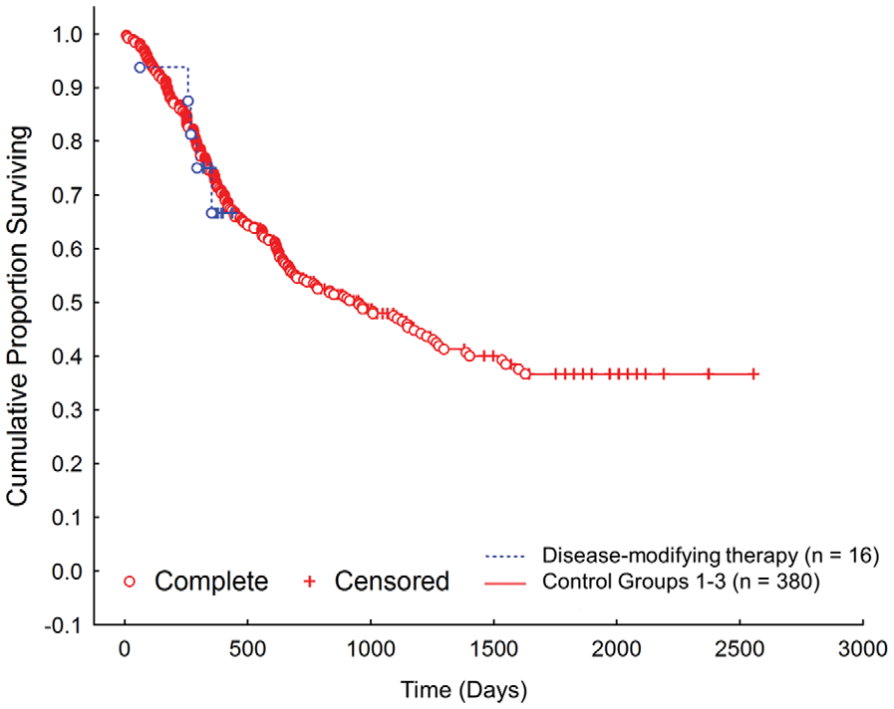
Contralateral cranial cruciate ligament (CrCL) survival after post-operative treatment with provisional disease-modifying therapy. Kaplan-Meier plot for contralateral CrCL survival in a population of client-owned dogs treatment with oral doxycycline after TPLO stabilization of unilateral CrCLR. Arthroscopic examination and associated lavage of the contralateral stable stifle, together with intra-articular hyaluronic acid and oral doxycycline did not significantly influence CrCL survival (*p* = 0.87). Complete – dogs that experienced contralateral CrCLR during the study period; Censored – dogs that did not experience contralateral CrCLR during the study period.

One dog died during the study period shortly before the one-year long-term follow-up recheck examination was due. Doxycycline and intra-articular hyaluronic acid treatment after arthroscopic joint examination did not significantly influence contralateral CrCL survival time to rupture, when compared to the control population of 380 dogs that did not receive the provisional disease-modifying therapy (*p* = 0.87, **Fig. 3**).

Clinical metrology data were obtained from 11 of 13 dogs that were re-examined. Overall, load score showed a significant improvement in mobility at one-year after surgery, when compared with mobility at the time of initial diagnosis (*p*<0.05). LOAD scores were 21±11 and 10±7 immediately before surgery and at long-term follow-up respectively.

Force-plate analysis-of-gait was performed in 10 of 15 dogs at long-term follow-up. In 8 dogs, the contralateral stifle was determined to be clinically stable at recheck, whereas in the remaining two dogs, contralateral CrCLR was diagnosed. In the dogs with stable contralateral stifles at recheck, no significance differences in Fz or VI were found between index and contralateral stifles during force-plate analysis-of-gait (*p*>0.05, **Table 2**); Fz was higher in the stable contralateral pelvic limb, with an index-contralateral difference in Fz of −4.6%. At follow-up, both of the dogs that developed subsequent contralateral CrCLR and underwent gait analysis exhibited reduced weight-bearing in the contralateral stifle (index-contralateral difference in Fz was 43.7%).

**Table 2:** Force-plate analysis-of-gait at long-term follow-up after surgical stabilization and hyaluronic acid/doxycycline treatment of stifle arthritis in dogs with unilateral cranial cruciate ligament rupture.

Parameter	Index stifle	Contralateral Stifle	Index-Contralateral difference (%)	Significance
Peak Vertical Force	46.4±8.9	48.5±11.9	−4.6	NS
Vertical Impulse	14.1±3.9	13.6±2.9	3.5	NS

**Note**: Data represent mean ± standard deviation. n = 8 dogs; NS – not significant (*p*>0.05).

### Stifle radiography in HA/doxycycline-treated dogs

Radiographic scores for overall change were significantly worse in the unstable index stifle, when compared to the stable contralateral stifle (*p*<0.05, **Table 3**). In the stable contralateral stifle, effusion was identified in 12 of 16 stifles (75%) and all index stifles, and arthritis was identified in 15 of 16 stifles (94%), and all index stifles. Although the scores for overall change became higher over time, these changes were not significant in both the index and stable contralateral stifle (*p*>0.12 and *p*>0.11 respectively). Synovial effusion was also significantly worse in the index stifle at diagnosis, when compared with the stable contralateral stifle (*p*<0.01), although this difference was not significant at the recheck visits. Scores for synovial effusion did not change significantly over time in both the index and stable contralateral stifle (*p*>0.78 and *p*>0.14 respectively).

**Table 3:** Radiographic signs of stifle arthritis and synovial effusion in dogs with unilateral cranial cruciate ligament rupture after surgical stabilization and hyaluronic acid/doxycycline treatment.

Composite Score [28]	Index stifle	Contralateral Stifle	Significance
Diagnosis	6.5 (4–10)	4.5 (0–8)	*p*<0.005
10 weeks after surgery	7 (4–8)	4 (2–7)	*p*<0.005
1 year after surgery	8 (6–8)	6 (5–7)	*p*<0.05
**Synovial Effusion ** [**28**]			
Diagnosis	2 (1–2)	1 (0–2)	*p*<0.01
10 weeks after surgery	2 (1–2)	2 (0–2)	NS
1 year after surgery	2 (1–2)	2 (1–2)	NS

Data represent median (range). Diagnosis, n = 16; 12 weeks after surgery, n = 15; 1 year after surgery, n = 8. NS – not significant (*p*>0.05). Changes in composite score and synovial effusion were not significantly different over time. One dog was excluded from the analysis at the 10 week recheck, because of development of contralateral CCLR at 67 days after surgery, 5 dogs were excluded at long-term follow-up.

Stress radiographic measurements supported the finding of clinical stability in the contralateral stifles at initial diagnosis. In the contralateral stifles that remained clinically stable over time, there were no significant differences in tibial translation ratio [median (range) at diagnosis of 0.025 (0.000–0.106), n = 11; 1 year follow-up ratio was 0.025 (0.00–0.048), n = 7]. However, in the 3 dogs that were determined to have experienced subsequent contralateral CrCLR at long-term follow-up visit, cranial tibial translation ratio was higher [0.075 (0.073–0.184)].

### Histologic synovitis and bacterial load in HA/doxycycline-treated dogs

**Figure 4:**
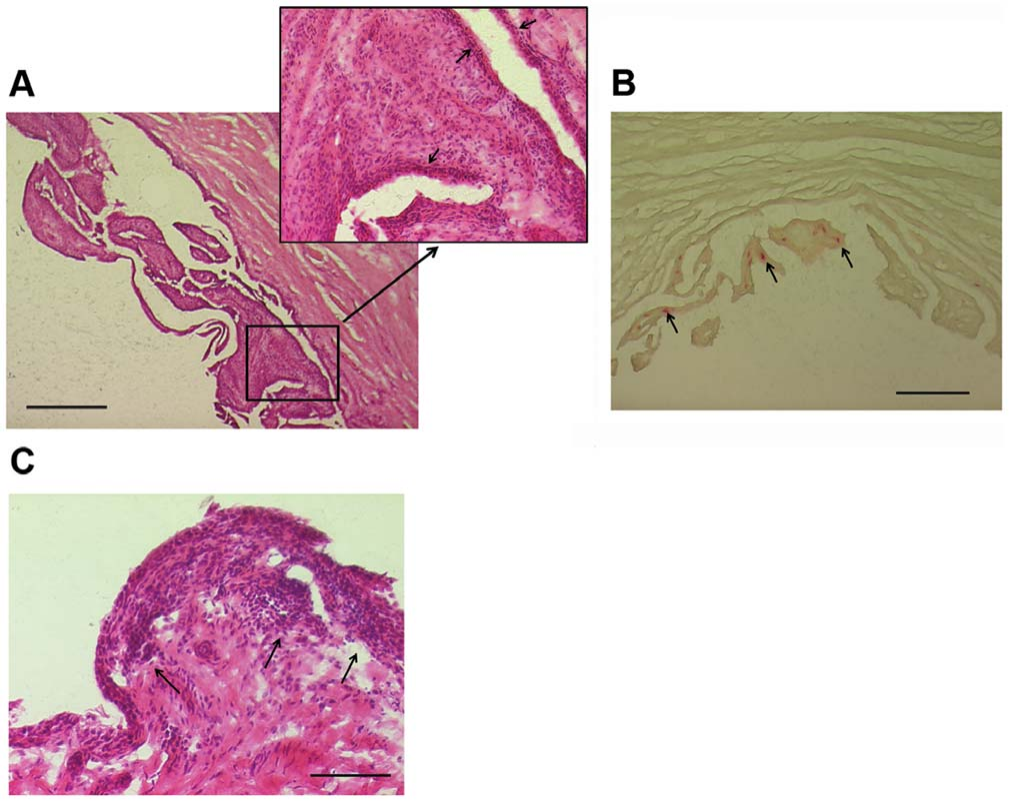
Photomicrographs of stifle synovium from dogs with unilateral cranial cruciate ligament rupture and a contralateral stable stifle. (**A**) Unstable stifle of a five-year-old neutered male Golden Retriever. Proliferation of the synovial intima with villus formation can be seen. Widening of intima (arrows) and infiltration of the intima and sub-intima with mononuclear inflammatory cells can also be seen. (**B**) Unstable stifle of a five-year-old Chesapeake Bay Retriever. TRAP^+^ mononuclear cells (arrows) were identified in synovial villi in the intima and sub-intimal tissues. (**C**) Stable stifle from a six-year-old neutered male Golden Retriever. Proliferation of the synovial intima can also be seen, with accumulation of mononuclear inflammatory cells within the intima and the sub-intimal tissues (arrows). A,C – Hematoxylin and eosin stain; B – histochemical stain for tartrate-resistant acid phosphatase (TRAP). A – bar = 500 µm; B – bar = 200 µm; C – bar = 100 µm.

Both unstable index and stable contralateral stifles had macroscopic and histologic evidence of synovitis. Synovitis assessed arthroscopically was significantly increased in the index stifle, when compared with the contralateral stable stifle (*p*<0.001, **Table 4**). Histologic synovitis was present in both stifles, with elevated severity scores in the unstable stifle; sub-intimal mononuclear inflammatory cells were often found (**Fig. 4**). Numbers of TRAP^+^ mononuclear cells were increased in the unstable index stifle, when compared with the contralateral stable stifle. TRAP^+^ mononuclear cells were identified in 11 of 14 unstable stifles, but only 2 of 10 stable stifles (**Fig. 4**).

**Table 4:** Synovial inflammation in the unstable index and stable contralateral stifles of dogs with unilateral cranial cruciate ligament rupture.

	Unstable Index Stifle	Stable Contralateral Stifle	Significance
NRS Arthroscopic Synovitis (n = 16)	19 (11, 22)	12 (4, 20)	***p*** **<0.001**
VAS Arthroscopic Synovitis (n = 16)	63±21	35±20	***p*** **<0.001**
Histologic inflammation (n = 12–14)	33±23	20±20	**NS**
TRAP^+^ mononuclear cells (n = 10–14)	19 (0, 42)	0 (0, 31)	***p*** **<0.05**

**Note**: Synovitis was scored subjectively using arthroscopy using a compartmental numerical rating scale (NRS, score range 0–24) and a visual analogue scale (VAS). Histologic sections of synovium were also subjectively graded for inflammation and numbers of TRAP^+^ mononuclear cells using a visual analogue scale; severity scores ranged from 0 (no inflammation) to 100 (could not be more severely inflamed). NS – not significant.

No significant changes in bacterial load were identified using the Nadkarni 16S rRNA universal primer set. With the Yang 16S rRNA universal primer set, bacterial load within synovial fluid was increased in the contralateral stifle versus the unstable index stifle at diagnosis (*p*<0.05, **Table 5**). At the 10-week recheck, bacterial load in synovial fluid in the unstable stifle was increased when compared to diagnosis (*p*<0.01, **Table 5**). Bacterial load in synovial fluid in the contralateral stable stifle was also higher at the 10-week recheck (*p* = 0.11).

**Table 5:** Bacterial load in the unstable index and stable contralateral stifles of dogs with unilateral cranial cruciate ligament rupture.

16S rRNA Universal Primer Set	Unstable Index Stifle			Stable Contralateral Stifle		
	*Diagnosis*		*10 Week Recheck*	*Diagnosis*		*10 Week Recheck*
	*Synovium*	*Synovial fluid*	*Synovial fluid*	*Synovium*	*Synovial fluid*	*Synovial fluid*
Yang et al., (2002) [32]	53 (8, 1124)	41 (1, 514)	459## (7, 7931)	93 (10, 392)	212* (1, 2474)	1021 (86, 3882)
Nadkarni et al., (2002) [31]	40 (5, 442)	13 (3, 98)	15 (2, 64)	45 (2, 589)	17 (3, 89)	15 (2, 46)

**Note**: Data represent median (range). Bacterial load represents estimated 16S rRNA amplicon number using universal primers and real-time PCR.

*p<0.05 versus contralateral joint.

## *p*≤0.01 versus same tissue at diagnosis. (n = 13 to 16 dogs per group).

### Synovial tissue gene expression in HA/doxycycline-treated dogs

Results are summarized in **Table 6**. Overall, expression of TCR Vβ and CD3ε were highly correlated (S_R_ = 0.79, *p*<0.0001). At diagnosis, expression of TCR Vβ, CD3ε, IL-10, IFN-γ, and TRAP in synovium was increased in the unstable index stifle, when compared with the stable contralateral stifle (*p*<0.05). Expression of TNF-α was increased in synovial fluid in the unstable index stifle, when compared with the stable contralateral stifle (*p*<0.01).

**Table 6:** Relative expression of immune response genes in synovial fluid from unstable index and stable contralateral stifles of dogs with unilateral cranial cruciate ligament rupture.

	Synovium	Synovial fluid		
Gene	Unstable Index Stifle			
	*Diagnosis*		*10 Week Recheck*	*1 Year Recheck*
TCR Vβ	** **0.03** (0.00, 3.33)	0.61 (0.01, 9.89)	0.64 (0.06, 13.8)	0.21 (0.05, 8.49)
CD3ε	* **0.02** (0.00, 2.47)	**0.69** (0.01, 3.08)	**0.20** (0.03, 2.24)	**0.1** (0.01, 1.24)
IL-17	**2.59** (0.16, 337)	**3.84** (0.04, 451)	1.76 (0.08, 232)	
IL-10	** **7.98** (0.21, 184)	**14.2** (0.00, 331)	## **55.0** (0.13, 1686)	
IL-4	1.10 (0.00, 35.4)	**4.64** (0.27, 112)	1.17 (0.01, 785)	
IFN-γ	**1.38 (0.01, 10480)	12.4 (0.01, 3572)	* **8.99** (0.51, 100)	
TRAP	**1.08 (0.01, 95.9)	**5.78** (0.48, 53.9)	** **24.4** (0.06, 124)	
TNF-α	0.11 (0.00, 5.39)	**2.06 (0.00, 18.6)	*1.39 (0.32, 14.9)	2.75 (0.20, 16.8)
	**Stable Contralateral Stifle**			
TCR Vβ	**0.02** (0.00, 0.18)	**0.11** (0.01, 43.03)	0.21 (0.00, 1711)	0.40 (0.07, 1.16)
CD3ε	**0.02** (0.00, 0.08)	**0.15** (0.01, 22.3)	**0.04** (0.00, 1270)	0.17 (0.05, 1.00)
IL-17	2.71 (0.00, 122)	**4.58** (0.05, 23347)	1.87 (0.00, 84)	
IL-10	1.00 (0.02, 18.0)	**17.0** (0.04, 98.3)	**29.0** (0.18, 250)	
IL-4	0.80 (0.01, 15.8)	2.11 (0.00, 153)	**17.1** (0.80, 139407)	
IFN-γ	0.33 (0.00, 761)	0.63 (0.02, 126)	0.73 (0.00, 137)	
TRAP	**0.11** (0.01, 2.92)	**2.28** (0.26, 36.6)	1.87 (0.03, 92.0)	
TNF-α	**0.04** (0.00, 0.42)	**0.31** (0.00, 9.41)	0.38 (0.00, 5.50)	1.10 (0.46, 6.39)

**Note**: TCR Vβ - beta chain of the canine T cell receptor; TRAP – tartrate-resistant acid phosphatase; IL – interleukin; IFN – interferon; TNF – tumor necrosis factor. Data represent median (range). Median values in bold indicate that gene expression is significantly different from the peripheral blood mononuclear cell internal control (*p*<0.05).

*p<0.05,

**p<0.01 versus contralateral stifle.

## *p*<0.01 versus same tissue at diagnosis. n = 15–16 at diagnosis, n = 14–15 at 10 week recheck, n = 5–7 at 1 year recheck.

At the 10-week recheck, expression of IFN-γ (*p*<0.05), TRAP (*p*<0.01), and TNF-α (*p*<0.05) in synovial fluid in the unstable index stifle was also higher, when compared with the stable contralateral stifle. Expression of IL-10 (*p*<0.01) in synovial fluid from the unstable index stifle was also increased, when compared to diagnosis. No significant differences in gene expression were detected at the one-year recheck.

### Correlative analyses in HA/doxycycline-treated dogs

Arthroscopic VAS synovitis severity score (r^2^ = 0.57, *p* = 0.005) and the presence of TRAP^+^ mononuclear cells in the stable stifle synovium (S_R_ = 0.63, *p* = 0.05) were significantly related to histologic synovitis score at diagnosis. No significant correlation was detected in the unstable index stifle. At diagnosis, bacterial load in synovium and synovial fluid was not significantly related to arthroscopic or histologic severity score, or numbers of TRAP^+^ mononuclear cells in either stifle. Bacterial load was not significantly correlated with histologic inflammation or synovial gene expression at diagnosis, except that bacterial copy number estimated using the Nadkarni primers was correlated with expression of TNF-α in synovial fluid in the stable contralateral stifle (S_R_ = 0.6, *p*<0.05). In the unstable stifle at diagnosis, histologic synovitis was inversely correlated with expression of IL-4 in synovium (S_R_ = −0.77, *p* = 0.001). Additionally, the presence of TRAP^+^ mononuclear cells in synovium was significantly correlated with IL-10 expression in synovium (S_R_ = 0.69, *p*<0.01). No significant correlations between the presence of a tear in the medial meniscus of the unstable index stifle and any marker of stifle joint inflammation were found.

Correlative analysis between a priori clinical variables and development of subsequent contralateral rupture revealed few significant results. Radiographic assessment of stifle arthritis and effusion, and arthroscopic assessment of synovial inflammation and CrCL fiber tearing were not significantly correlated with subsequent contralateral CrCLR. Pre-operative TPA was not significantly correlated with development of subsequent contralateral CrCLR.

At diagnosis, histologic inflammation in the unstable (S_R_ = 0.39, *p* = 0.17) and stable stifle (S_R_ = 0.47, *p* = 0.13) was not significantly related to subsequent contralateral rupture. Expression of CD3ε in synovium in the unstable stifle was significantly related to subsequent contralateral CrCLR (S_R_ = 0.60, *p* = 0.01), although this was not significant for the stable contralateral stifle. There was also a similar trend for expression of TCR Vβ and TRAP in unstable stifle synovium (S_R_ = 0.54, *p* = 0.03 and S_R_ = 0.52, *p* = 0.05 respectively). Expression of IL-17 in synovial fluid in the stable stifle was inversely correlated with development of subsequent contralateral CrCLR (S_R_ = −0.65, *p* = 0.01). Correlations with IL-10, IL-4, IFN-γ, and TNF-α were not significant.

## Discussion

It has been recognized for some time that dogs affected with CrCLR often ultimately develop bilateral ruptures. Bilateral rupture may be diagnosed at initial presentation, or by development of subsequent contralateral CrCLR, often within a relatively short period of time from diagnosis. Risk of subsequent contralateral CrCLR has typically been reported as a proportion at long-term follow-up, often around one year from diagnosis [2], [3], [5]–[7]. This risk is in the range of 22–54% [2], [3], [5]–[7]. In the present study, this risk was 54% and was remarkably similar in the different cohorts within the study. Median survival time to subsequent contralateral CrCLR in our large baseline population of dogs treated surgically for unilateral rupture was 947 days overall. Interestingly, the Kaplan-Meier survival plot yielded an exponential shaped curve, particularly for Groups 1 and 2, which had the longest follow-up periods. The shape of the survival curve suggests that within the overall population, there are individual dogs that are protected from the trait, with a long contralateral CrCL survival time, as well as individual dogs that are susceptible to the trait and more rapidly experience subsequent contralateral CrCLR. The factors that might influence this susceptibility are not fully understood. However, the presence of stifle synovitis histologically increases the risk of subsequent contralateral CrCLR [10], although there is no evidence of a MHC class II immunogenetic association [38]. Non-contact CrCLR is a complex trait, with multiple genes contributing to development of the phenotype [39]. Heritability is estimated to be 0.27 in Newfoundlands, suggesting that extrinsic factors also have a substantial effect on expression of the phenotype [40].

Both intrinsic and extrinsic factors are thought to contribute to the risk of developing CrCLR. Intrinsic factors include genetics, cruciate ligament matrix composition, stifle morphology, and body weight. Extrinsic factors include body condition, whether or not ovariohysterectomy or castration has been performed, and development of synovitis [4], [10], [39]–[45]. In the present study, we examined the effect of gender, breed, body weight, age, and TPA on risk of subsequent contralateral CrCLR in a large population of dogs. The effects of breed and body weight were not significant. However, we found that TPA significantly influenced the risk of contralateral CrCLR, suggesting that stifle morphology and functional loading of the CrCL during daily life is a risk factor for CrCLR, although this was not confirmed in our smaller cohort of dogs treated with HA/doxycycline. Although stifle morphological factors appear to confer some risk of CrCLR, stifle morphology alone and TPA, in particular, does not appear to be a primary causative factor [46]. The age range when CrCLR develops is typically between 2–10 years [43], [44]. In the present study, we found that age significantly influenced CrCL survival, with increasing age being associated with increased contralateral CrCL survival, although this effect also appeared small in magnitude. This observation also fits with the concept that our overall study population consisted of dogs with a mixture of intrinsic (genetic) susceptibility to the CrCLR trait. Neutered dogs also had shortened contralateral CrCL survival, as previously described [43], [44].

In a second experiment, we examined long-term CrCL survival in a small cohort of dogs with unilateral CrCLR that were treated with HA/doxycycline and surgical stabilization of the index stifle. We did not find a significant treatment effect with our provisional disease-modifying therapy. Correlative analysis revealed few significant associations between markers of stifle disease and subsequent development of contralateral CrCLR, although increased expression of T-cell associated genes in the unstable stifle at diagnosis was correlated with development of subsequent contralateral CrCLR. This observation fits with the concept that the presence of stifle synovitis is related to the risk of subsequent contralateral CrCLR [10], although the correlation between expression of IL-17 in synovial fluid in the stable stifle and development of subsequent contralateral CrCLR that we detected was an inverse one. Further work is needed studying association of the trait with MHC genotype, although current evidence suggests a strong class II association is unlikely [38]. Bacterial load was not significantly correlated with histologic synovitis, although increased copy number was detected in the unstable stifle, which increased over time. Although we have previously reported association and weak correlation between bacterial load and stifle synovitis/arthritis in CrCLR dogs [22]–[24], collectively these results suggest that bacterial load is not a key factor in development of stifle synovitis in affected dogs.

In this proof-of-principle study, we selected the test compounds used in the trial based on existing literature in the field of disease-modifying therapy, including both anti-inflammatory compounds, as well as compounds that may ameliorate connective tissue matrix degradation over time. Additionally, both of the treatments used are generic and inexpensive. Doxycycline has been widely studied as a potential therapeutic drug for arthritis. In-vitro, doxycycline can inhibit gelatinase-mediated degradation of type XI collagen, reduce activation of collagenase, and inhibit mRNA for inducible nitric oxide, a mediator of tissue catabolism [26], [47], [48]. In-vivo studies in dogs suggest that treatment can modify development of arthritis over time [27], using a treatment regimen of similar duration and dosage to that of the present study. Indirectly, this may suggest that disease-modifying therapy that targets synovial inflammation may be a more effective treatment strategy than therapy that targets ligament matrix degeneration, as the doxycycline regimen used in this study did not significantly influence CrCL survival. An additional point supporting this concept is the recent observation that synovitis is an early event in canine non-contact CrCL rupture, which precedes development of arthroscopically visible fraying of the cruciate ligament complex [8].

HA has also been widely studied as a disease-modifying treatment for arthritis both as a course of intra-articular injections, as well as using a single injection protocol [49], [50]. Potential therapeutic effects on arthritic joints from intra-articular HA injection are complex and include reduction in both synovial inflammation, and connective tissue matrix degeneration through inhibition of MMP-13 expression, for example [51]. Pro-inflammatory effects of HA fragmentation may occur through CD44 signaling [52]. However, treatment with intra-articular HA did not reduce CD44 expression in OA joints experimentally in an ovine meniscectomy model, but did reduce synovial vascularity and subintimal fibrosis [49]. In the present study, we also used arthroscopy to examine both the index and contralateral stifles at initial diagnosis [8]. The joint lavage associated with the arthroscopic procedure has the potential to exert a disease-modifying effect, by reducing synovial inflammation and production of pro-inflammatory cytokines, such as TNF-α [53].

In the dogs in this cohort, overall mobility was significantly improved at long-term follow-up, with equalization of weight-bearing between the index stifle treated with TPLO and the contralateral stifle, except for the dogs that developed subsequent contralateral CrCLR during the study period. Radiographic signs of arthritis were significantly worse in the unstable index stifle. There was also some evidence of worsening synovial effusion in the stable contralateral stifle over time, although no clinical or radiographically detectable changes in stability were identified. Synovial effusion was significantly worse in the index stifle at diagnosis, but not at the follow-up visits. Similarly, there was little change in expression of gene markers for T cells and macrophages over time. Increases in expression of the T-cell associated genes TCR Vβ, CD3ε, and IFN-γ that were identified in the unstable index stifle relative to the contralateral stable stifle were principally identified at diagnosis, although expression of IFN-γ remained increased in the unstable stifle at the 10 week recheck. Up-regulation of macrophage-associated genes (IL-10, TRAP, and TNF-α) was also identified in the unstable stifle at diagnosis and also at the 10-week recheck; there was no significant difference in TNF-α between index and contralateral stifles at the one-year recheck. These observations suggest that development of stifle synovitis may be mediated via T lymphocyte and macrophage signaling, which was not influenced by oral doxycycline treatment.

Interestingly, IL-10 was the only gene whose expression was increased in synovial fluid from the unstable stifle at the 10-week recheck. Relative expression was also higher in the contralateral stable stifle. IL-10 is a cytokine with anti-inflammatory properties that is typically up-regulated in arthritic joints [54], [55]. Up-regulation of IL-10 in both stifles relative to internal control, with increased expression over time suggests that immune regulating cells, such as macrophages and regulatory T cells, are acting to inhibit effector T cells and activated macrophages, and suppress synovial inflammation in affected joints. In humans with rheumatoid arthritis, there is impairment of regulatory T cell function [56]. Polymorphisms in the promotor region of IL-10 are known to influence the risk of rheumatoid arthritis in human beings, by reducing IL-10 transcription and decreasing circulating IL-10 [57]. Similarly, IL-10 polymorphisms have been associated with immune-mediated disease in the dog [58]. Whether there is functional impairment of anti-inflammatory cytokine responses in dogs with stifle synovitis and non-contact CrCL rupture has not been determined. In the present study, the presence of TRAP^+^ mononuclear cells in synovium was significantly correlated with IL-10 expression in the unstable stifle at diagnosis, supporting the concept that IL-10 production by activated macrophages may be a functionally important anti-inflammatory factor within the joint environment, acting to ameliorate synovitis in this disease.

Although we did not identify an obvious treatment effect on CrCL survival, or significant progression of arthritis pathology over time, the lack of a treatment effect from arthroscopic lavage and associated medical therapy suggests that this in-vivo client-owned canine model of unilateral CrCLR (with contralateral incipient disease) may be valuable for future mechanistic and therapeutic studies, since pathogenesis of naturally occurring non-contact CrCLR in client-owned dogs is substantially different from traumatic CrCLR modeled by CrCL transection in the Pond-Nuki model [59], and has many similarities to the non-contact minimal trauma ACL rupture that is common in human beings, particularly women [60], [61].

The lack of a significant treatment effect in this cohort, with a 31% contralateral rupture rate at one year, also suggests that our arthroscopic findings in the stable stifle at diagnosis are a valid reflection of incipient disease. Arthroscopic grading of synovitis has been widely performed in human beings with arthritic conditions [62], but not in dogs [8]. All client-owned dogs had medical treatment with non-steroidal anti-inflammatory drugs of variable duration before inclusion in this study [8], but this medical therapy did not prevent development of subsequent contralateral CrCLR in the cohort.

This study had several limitations. A potential concern with our ligament survival analysis was that limited phenotypic and clinical status data were available for each dog. It is common for CrCLR-affected dogs to be treated with a non-steroidal anti-inflammatory drug (NSAIDs). Whether NSAID medication has any effect on CrCL survival could not be determined from the present study. Given that stifle synovitis increases the risk of subsequent contralateral CrCLR in client-owned dogs [10], in future work it would be interesting to determine, in more detail, whether or not synovitis and the presence of specific T cell subsets in joint tissues or peripheral blood [63] have a significant effect on contralateral CrCL survival over time. Although difficult to obtain in a client-owned dog model, follow-up second-look arthroscopy and synovial biopsies may have also yielded additional data on synovitis status over time. Several dogs in the treatment cohort developed subsequent contralateral rupture during the study period. Collection and analysis of additional stifle joint tissue samples in these dogs at the time of contralateral stifle stabilization surgery may also have been informative. More detailed study of the phenotype of the inflammatory cells within the synovium of stable stifles [63] and analysis of other biomarkers of joint inflammation, such as C-reactive protein [64], may also have yielded additional data on the relationship between synovitis and development of non-contact CrCLR. In addition, mobility questionnaire data were obtained retrospectively at long-term follow-up. Although efficacy of intra-articular HA has been described after use of a single injection [50], treatment is often given as a course of injections [49]. Efficacy of HA treatment may have been improved with a course of intra-articular injections, although risk of adverse effects may also be increased.

In conclusion, subsequent contralateral CrCLR is common in client-owned dogs, with a median ligament survival time of 947 days. Treatment with arthroscopic lavage, single intra-articular HA injection, and oral doxycycline does not significantly influence contralateral CrCL survival. In this client-owned model of stifle arthritis and non-contact minimal trauma CrCLR, development of cranial tibial translation is preceded by development of synovial inflammation, superficial fraying of the cruciate ligament complex, and rupture of ligament fibers, particularly within the caudolateral bundle of the CrCL [8]. The immunological mechanism that induces development of synovitis in stable stifles with incipient CrCLR should be the focus of future work on identification of targets for disease-modifying therapy. Here T cell-mediated synovitis appears important. More work is also needed on identification of a genetic marker or a clinically relevant biomarker that is a good predictor of progressive ligament rupture over time.
